# Non-linear association between metabolic score for insulin resistance and diabetes mellitus in normal-weight middle-aged and older Chinese adults: a multicenter retrospective cohort study

**DOI:** 10.3389/fnut.2025.1717792

**Published:** 2026-01-20

**Authors:** Hualang Cai, Zhimao Cai, Qingxian Cai

**Affiliations:** 1 Department of Hepatology, National Clinical Research Center for Infectious Diseases, The Third People's Hospital of Shenzhen, Shenzhen, Guangdong, China; 2 Shenzhen University Health Science Center, Shenzhen, Guangdong, China; 3 Department of General Medicine, Shenzhen Second People's Hospital, The First Affiliated Hospital of Shenzhen University, Shenzhen, Guangdong, China

**Keywords:** cohort study, diabetes mellitus, insulin resistance, METS-IR, middle-aged and older, normal-weight

## Abstract

**Background:**

Although the link between the metabolic score for insulin resistance (METS-IR) and diabetes mellitus (DM) is well-established in general populations, it remains underexplored in normal-weight middle-aged and old adults—an often-overlooked group in metabolic research. Therefore, this study investigated the association between METS-IR and diabetes in this specific population.

**Methods:**

A retrospective cohort study was conducted using data from the Rich Healthcare Group database, including 23,692 normal-weight (body mass index: 18.5–23.9 kg/m^2^) Chinese adults aged ≥45 years. Cox proportional hazards models were applied with adjustments for demographic, clinical, and biochemical confounders. Non-linearity was examined using smooth curve fitting and threshold effect analysis.

**Results:**

Higher METS-IR values were significantly correlated with greater diabetes risk during follow-up. In the model 3, each 1-unit rise in METS-IR corresponded to a 12% increase in diabetes risk (HR: 1.12, 95% CI: 1.10, 1.14). The analysis revealed a non-linear association, with a critical inflection point at a METS-IR of 37.24. Below this threshold, risk rose more sharply (HR: 1.18, 95% CI: 1.14, 1.21 per unit), whereas above the threshold, the association was attenuated (HR: 0.96, 95% CI: 0.89, 1.04). Subgroup analyses showed largely consistent findings across strata.

**Conclusions:**

Among normal-weight middle-aged and older Chinese adults, METS-IR exhibits a positive, non-linear relationship with DM risk. Maintaining a lower METS-IR may help prevent the development of DM.

## Introduction

Diabetes mellitus (DM) poses a substantial and escalating global health burden, with its prevalence increasing rapidly, especially among aging populations (1). According to 2021 estimates by the International Diabetes Federation, approximately 537 million adults (20–79 years) had diabetes, a number expected to rise to 783 million by 2045. This steep increase underscores the critical need for effective public health strategies (2, 3). Such a progressive increase in diabetes cases also has major socioeconomic consequences, including rising healthcare costs and loss of productivity, marking DM as one of the most pressing health issues of the 21st century (4). A key pathophysiological feature preceding type 2 DM (T2DM) is insulin resistance (IR), which typically presents as diminished cellular responsiveness to insulin, due to chronic inflammation, excess adiposity, and metabolic dysfunction (5–7). Although obesity, particularly visceral fat accumulation, is a well-documented risk factor contributing to IR and subsequently the development of T2DM, it is essential to note that a significant proportion of individuals with diabetes maintain a normal body weight, particularly among middle-aged and older adults (8, 9). T2DM and non-alcoholic fatty liver disease (NAFLD) are closely linked in lean individuals (10). On the other hand, normal-weight individuals, including middle-aged and elderly adults, can develop NAFLD, and it may even present with more severe liver disease and higher mortality risks compared to obese individuals. This condition, known as lean NAFLD, is often overlooked because it lacks the typical signs of obesity (11). This pattern reinforces the notion that relying solely on body mass index (BMI) for metabolic risk assessment is inadequate.

Calculated based on BMI high-density lipoprotein cholesterol (HDL-C), triglyceride (TG), and fasting plasma glucose (FPG), the metabolic score for IR (METS-IR) serves as a useful non-insulin-based surrogate for assessing IR (12, 13). This composite index provides a cost-effective and accessible tool, especially beneficial in large-scale epidemiological studies where comprehensive insulin assays may be unfeasible (14). METS-IR is known to correlate significantly with several pathological components of IR, including visceral and intrahepatic fat content (15), thus highlighting its utility in identifying at-risk individuals even before diabetes becomes clinically evident (16). Notably, METS-IR has been associated with an increased risk of diabetes in a wide range of populations, including those with varying body weights (17). However, greater attention must be directed toward the often-overlooked normal-weight demographic, as evidence suggests that these individuals still harbor metabolic abnormalities (18, 19). Normal-weight middle-aged and elderly adults can still have increased carotid intima-media thickness (CIMT) if they have other risk factors like central obesity, high blood pressure, or high blood sugar (20), importantly, CIMT and NAFLD remain closely associated, contributing substantially to cardiovascular disease risk (21).

This is clinically significant because normal-weight adults, particularly those in middle-age or older, often show latent metabolic dysfunction despite having a BMI in the normal range. Age-related changes, including sarcopenia and visceral fat accumulation, predispose these adults to IR without overt obesity (22, 23). However, there is a paucity of data on the link between METS-IR and diabetes risk in normal-weight middle-aged and older adults. Therefore, we leveraged a large retrospective cohort from China's Rich Healthcare Group to examine the link between METS-IR and DM in a cohort focused specifically on normal-weight adults aged ≥45 years. Our findings are expected to inform early preventive strategies tailored to normal-weight individuals who may be overlooked in routine diabetes screening, and could also improve understanding of their broader metabolic health, including NAFLD and cardiovascular risk.

## Methods

### Study design and population

This secondary analysis used data from a publicly available cohort of 211,833 Chinese adults originally compiled by Chen et al. (24), and obtained from the Dryad Digital Repository (https://datadryad.org/stash/dataset/ doi: 10.5061/dryad.ft8750v). The dataset includes detailed medical records derived from health examination programs. As an open-access repository, Dryad permits the free download and reuse of data for secondary analyses in accordance with its terms of service. The original study received ethical approval from the Rich Healthcare Group Review Board, and thus no additional review was required for this secondary analysis. Both the original investigation and the present analysis adhered to the ethical principles of the Declaration of Helsinki.

**Figure 1:**
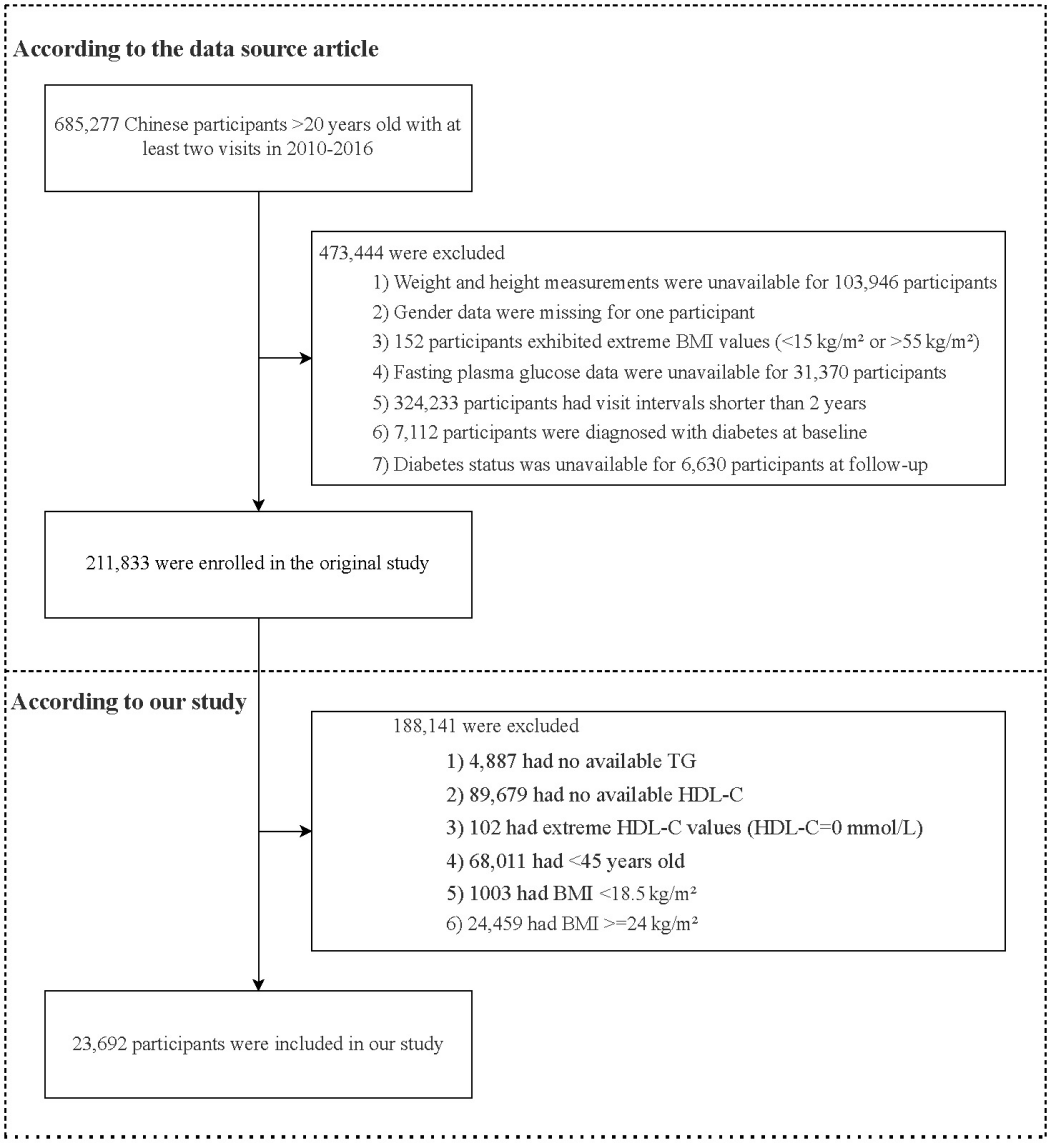
Participants selection flowchart. BMI, body mass index; TG, triglyceride; HDL-C, high-density lipoprotein cholesterol.

The baseline population was drawn from 685,277 Chinese adults (≥20 years) who completed health screenings between 2010 and 2016. After applying standard exclusions for missing data, extreme values, baseline diabetes, and insufficient follow-up, the final dataset contained 211,833 individuals. For the current analysis of normal-weight middle-aged and elderly adults, we further excluded participants aged < 45 years, those with missing triglyceride or HDL-C measurements, extreme HDL-C values (HDL-C = 0 mmol/L), and BMI outside the normal-weight range (< 18.5 or ≥24 kg/m^2^), resulting in a final analytical sample of 23,692 participants (Figure 1).

### METS-IR evaluation

The METS-IR value for each participant was calculated based on their clinical characteristics using the following formula: METS-IR = Ln [(2 × FPG (mmol/L) × 18) + TG (mmol/L) × 88.5] × BMI (kg/m^2^)/Ln [HDL-C (mmol/L) × 38.7] (12).

### DM assessment

DM was defined as FPG ≥ 7.00 mmol/L and/or a self-reported physician diagnosis. Follow-up for each participant was terminated upon diabetes diagnosis or at their last visit, whichever occurred first (24, 25).

### Data collection

As described previously (24), trained research staff, who completed standardized protocol training before data collection, performed all procedures to ensure accuracy and consistency. Data were collected on demographic characteristics, including smoking status, age, family history of diabetes, gender, and drinking status. Physical examinations included standardized measurements of height, weight, and blood pressure. After at least a 10-h fast, venous blood samples were obtained from all participants. Biochemical parameters—total low-density lipoprotein cholesterol (LDL-C), total cholesterol (TC), FPG, TG, serum creatinine (Scr), alanine aminotransferase (ALT), HDL-C, and blood urea nitrogen (BUN)—were analyzed using an automated biochemistry analyzer (Beckman 5800).

### Statistical analysis

In this study, baseline characteristics of the study population were first described in detail. Continuous variables were presented as means ± standard deviation, while categorical variables were summarized as frequencies and percentages. Survival rates and cumulative event incidence were estimated using the Kaplan–Meier method.

Cox proportional hazards regression was performed to quantify the relationship between METS-IR and incident diabetes, generating hazard ratios (HRs) and 95% confidence intervals (CIs). Three sequentially adjusted models were constructed: Model 1 remained unadjusted; Model 2 was adjusted for diastolic blood pressure (DBP), age, systolic blood pressure (SBP), and gender; and Model 3 was further adjusted for LDL-C, family history of diabetes, BUN, TC, Scr, and ALT. To validate the findings from the continuous METS-IR variable and assess trends, METS-IR was categorized into quartiles, and *P*-value for trend was calculated.

To examine potential non-linearity in the METS-IR-DM relationship, a Cox proportional hazards model with smooth curve fitting (cubic splines) was applied for graphical assessment. When non-linearity was detected, the inflection point was identified using a recursive algorithm, followed by construction of a piecewise Cox model. A likelihood ratio test was then applied to compare the linear and non-linear models and to determine optimal fit (26).

To assess the robustness of the primary results, stratified and sensitivity analyses were performed. Stratified analyses were conducted across subgroups defined by gender, age, and family history of diabetes. Subgroup analyses by smoking and drinking status were not performed due to insufficient data resulting from a high degree of missing values. In the primary regression models, missing data were handled using complete-case analysis. The robustness of the findings was evaluated through two sensitivity analyses: first, by including variables with high missing rates (smoking and drinking status) without imputation; and second, by assigning dummy variables to all covariates with more than 5% missing values, including smoking and drinking status. Statistical analyses were performed using R and EmpowerStats, with significance defined as *P* < 0.05.

## Results

### Characteristics of study participants

Baseline characteristics of the 23,692 enrolled normal-weight middle-aged and older participants, stratified by METS-IR quartiles (Q1–Q4, *n* = 5,923 each), are shown in Table 1. With rising METS-IR quartile, participants tended to be older and displayed progressive increases in SBP, DBP, ALT, Scr, FPG, TG, and BMI, whereas HDL-C levels decreased. The proportions of current smokers, males, and current drinkers also rose across higher METS-IR quartiles.

**Table 1:** The baseline characteristics of participants.

**METS-IR quartile**	**Q1 (20.73–28.64)**	**Q2 (28.64–31.04)**	**Q3 (31.04–33.42)**	**Q4 (33.42–60.82)**	***P*-value**
Participants	5,923	5,923	5,923	5,923	
Age (years)	55.33 ± 9.21	55.96 ± 9.25	56.40 ± 9.18	57.19 ± 9.08	< 0.001
SBP (mmHg)	117.55 ± 17.24	120.21 ± 17.70	122.52 ± 17.25	125.02 ± 17.71	< 0.001
DBP (mmHg)	72.66 ± 10.44	74.24 ± 10.76	75.74 ± 10.65	77.69 ± 10.71	< 0.001
ALT (U/L)	17.46 ± 23.07	18.92 ± 25.32	20.00 ± 12.64	23.75 ± 18.44	< 0.001
BUN (mmol/L)	4.80 ± 1.27	4.84 ± 1.23	4.88 ± 1.22	4.88 ± 1.21	0.001
Scr (mmol/L)	64.49 ± 14.64	66.91 ± 14.97	69.89 ± 15.33	72.55 ± 16.12	< 0.001
LDL-C (mmol/L)	2.89 ± 0.67	2.91 ± 0.69	2.95 ± 0.68	2.93 ± 0.75	< 0.001
TC (mmol/L)	5.07 ± 0.88	5.00 ± 0.91	4.99 ± 0.91	5.00 ± 0.96	< 0.001
BMI (kg/m^2^)	20.27 ± 1.02	21.67 ± 1.02	22.48 ± 0.93	23.00 ± 0.75	< 0.001
FPG (mmol/L)	4.82 ± 0.58	4.95 ± 0.58	5.05 ± 0.59	5.18 ± 0.64	< 0.001
TG (mmol/L)	0.87 ± 0.35	1.06 ± 0.46	1.30 ± 0.59	2.09 ± 1.33	< 0.001
HDL-C (mmol/L)	1.70 ± 0.33	1.51 ± 0.25	1.37 ± 0.21	1.15 ± 0.20	< 0.001
Gender					< 0.001
Male	1,668 (28.16%)	2,217 (37.43%)	2,900 (48.96%)	3,721 (62.82%)	
Female	4,255 (71.84%)	3,706 (62.57%)	3,023 (51.04%)	2,202 (37.18%)	
Smoking status					< 0.001
Current smoker	242 (19.01%)	314 (22.66%)	356 (25.25%)	568 (34.53%)	
Ever smoker	15 (1.18%)	36 (2.60%)	42 (2.98%)	64 (3.89%)	
Never smoker	1,016 (79.81%)	1,036 (74.75%)	1,012 (71.77%)	1,013 (61.58%)	
Drinking status					< 0.001
Current drinker	29 (2.28%)	36 (2.60%)	44 (3.12%)	67 (4.07%)	
Ever drinker	114 (8.96%)	162 (11.69%)	211 (14.96%)	259 (15.74%)	
Never drinker	1,130 (88.77%)	1,188 (85.71%)	1,155 (81.91%)	1,319 (80.18%)	
Family history of diabetes					0.647
No	5,785 (97.67%)	5,788 (97.72%)	5,804 (97.99%)	5,794 (97.82%)	
Yes	138 (2.33%)	135 (2.28%)	119 (2.01%)	129 (2.18%)	
Diabetes mellitus					< 0.001
No	5,847 (98.72%)	5,838 (98.56%)	5,778 (97.55%)	5,620 (94.88%)	
Yes	76 (1.28%)	85 (1.44%)	145 (2.45%)	303 (5.12%)	

Results are presented as Mean ± SD for continuous variables and N (%) for categorical variables. METS-IR, metabolic score for insulin resistance; SBP, systolic blood pressure; DBP, diastolic blood pressure; BMI, body mass index; TC, total cholesterol; TG, triglycerides; HDL-C, high-density lipoprotein cholesterol; LDL-C, low-density lipoprotein cholesterol; ALT, alanine aminotransferase; Scr, serum creatinine; BUN, blood urea nitrogen; FPG, fasting plasma glucose. Among the 23,692 participants, SBP had 6(0.03%) missing values, DBP had 6(0.03%) missing values, LDL-C had 59(0.25%) missing values, ALT had 83(0.35%) missing values, BUN had 637(2.69%) missing values, Scr had 200(0.84%) missing values, smoking status had 17,978 (75.88%) missing values, and drinking status had 17,978 (75.88%) missing values. In the primary regression models, missing data were handled using complete-case analysis.

### Tendency of DM across METS-IR quartiles in normal-weight middle-aged and older Chinese adults

**Figure 2:**
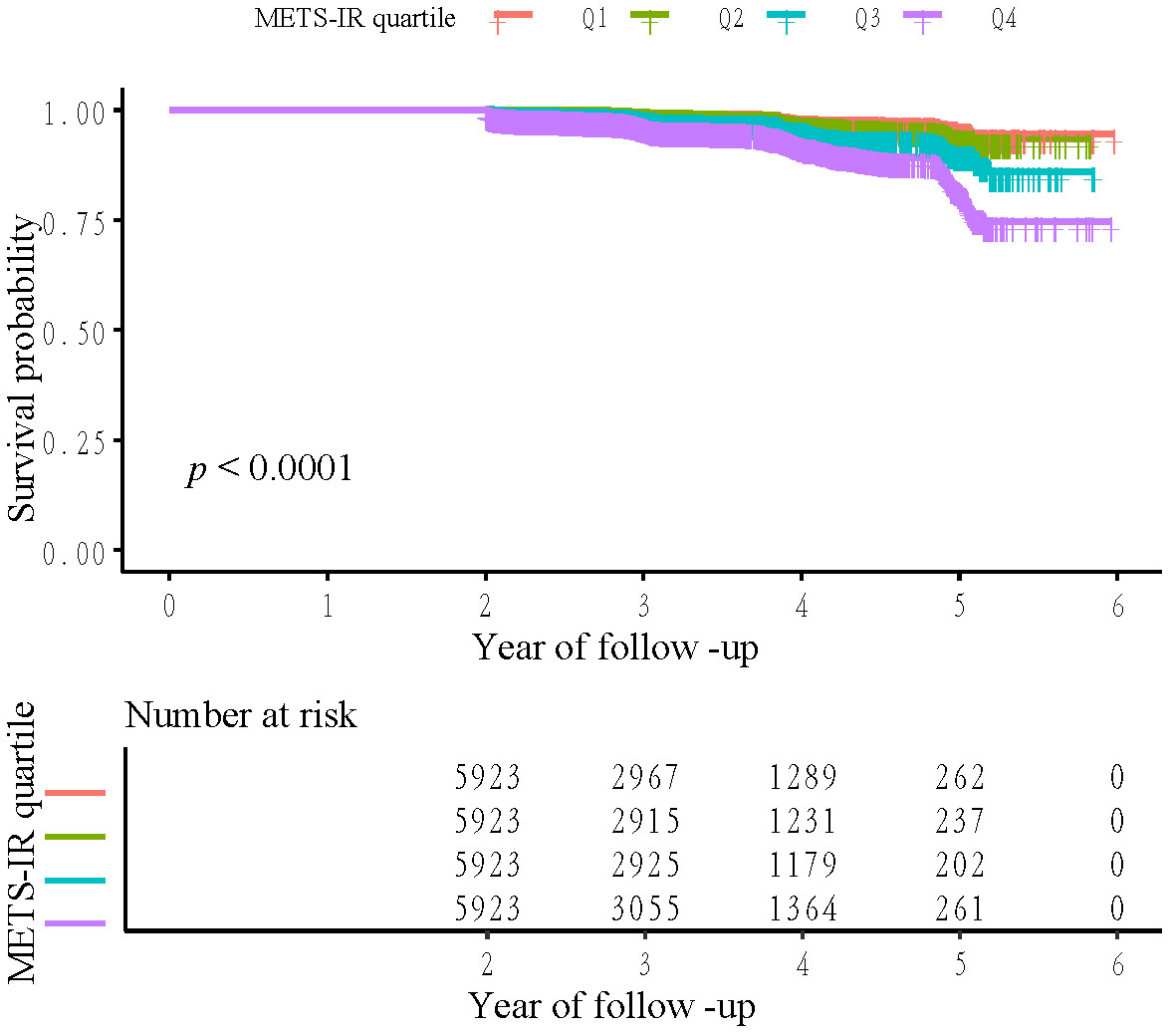
Kaplan–Meier event-free survival curve. Kaplan-Meier analysis of incident diabetes mellitus based on METS-IR quartiles in normal-weight middle-aged and older Chinese adults (log-rank, *P* < 0.0001).

Kaplan**–**Meier survival curves showing cumulative diabetes-free survival across METS-IR quartiles are presented in Figure 2. The curves are clearly separated, indicating a significant association between higher METS-IR and reduced diabetes-free survival (log-rank test, *p* < 0.0001). Participants in the highest quartile (Q4) exhibited the lowest survival probability, whereas those in the lowest quartile (Q1) maintained the highest probability throughout the follow-up period. The sample counts at each follow-up interval, indicated below the figure, clearly show a steady attrition of participants over time, which aligns with the Kaplan-Meier survival curve.

### Relationship of METS-IR with DM in normal-weight middle-aged and older Chinese adults

A significant positive association was observed between METS-IR and DM in normal-weight middle-aged and older Chinese adults (Table 2). In the Model 3, each 1-unit increase in METS-IR corresponded to a 12% higher diabetes risk (HR 1.12, 95% CI 1.10, 1.14, *P* < 0.0001). Quartile analyses showed a clear dose-response relationship (*P* for trend < 0.0001). Participants in the highest quartile (Q4) had a substantially elevated risk (HR 3.34, 95% CI 2.56, 4.36) compared to those in the lowest quartile (Q1) in Model 3. A significant risk increase was also evident for the third quartile (Q3; HR 1.86, 95% CI 1.39, 2.49), whereas the risk for the second quartile (Q2) remained non-significant. This graded association was consistent across progressively adjusted models.

**Table 2:** Association between METS-IR and diabetes mellitus in normal-weight middle-aged and older Chinese adults.

**Exposure**	**Model 1 (HR, 95% CI, *P*)**	**Model 2 (HR, 95% CI, *P*)**	**Model 3 (HR, 95% CI, *P*)**
METS-IR	1.13 (1.11, 1.15) < 0.0001	1.12 (1.10, 1.14) < 0.0001	1.12 (1.10, 1.14) < 0.0001
METS-IR quartile			
Q1	Ref.	Ref.	Ref.
Q2	1.17 (0.86, 1.59) 0.3319	1.06 (0.78, 1.45) 0.7130	1.11 (0.81, 1.54) 0.5073
Q3	2.03 (1.54, 2.69) < 0.0001	1.73 (1.31, 2.29) 0.0001	1.86 (1.39, 2.49) < 0.0001
Q4	3.92 (3.05, 5.04) < 0.0001	3.12 (2.41, 4.04) < 0.0001	3.34 (2.56, 4.36) < 0.0001
*P* for trend	< 0.0001	< 0.0001	< 0.0001

Model 1: unadjusted. Model 2: Model 1 +gender, age, SBP and DBP. Model 3: Model 2 + family history of diabetes, TC, LDL-C, Scr, BUN, and ALT; HR, hazard ratios; CI, confidence interval; Ref, reference. Missing data were handled using complete-case analysis.

### Threshold effect of METS-IR on DM in normal-weight middle-aged and older Chinese adults

**Figure 3:**
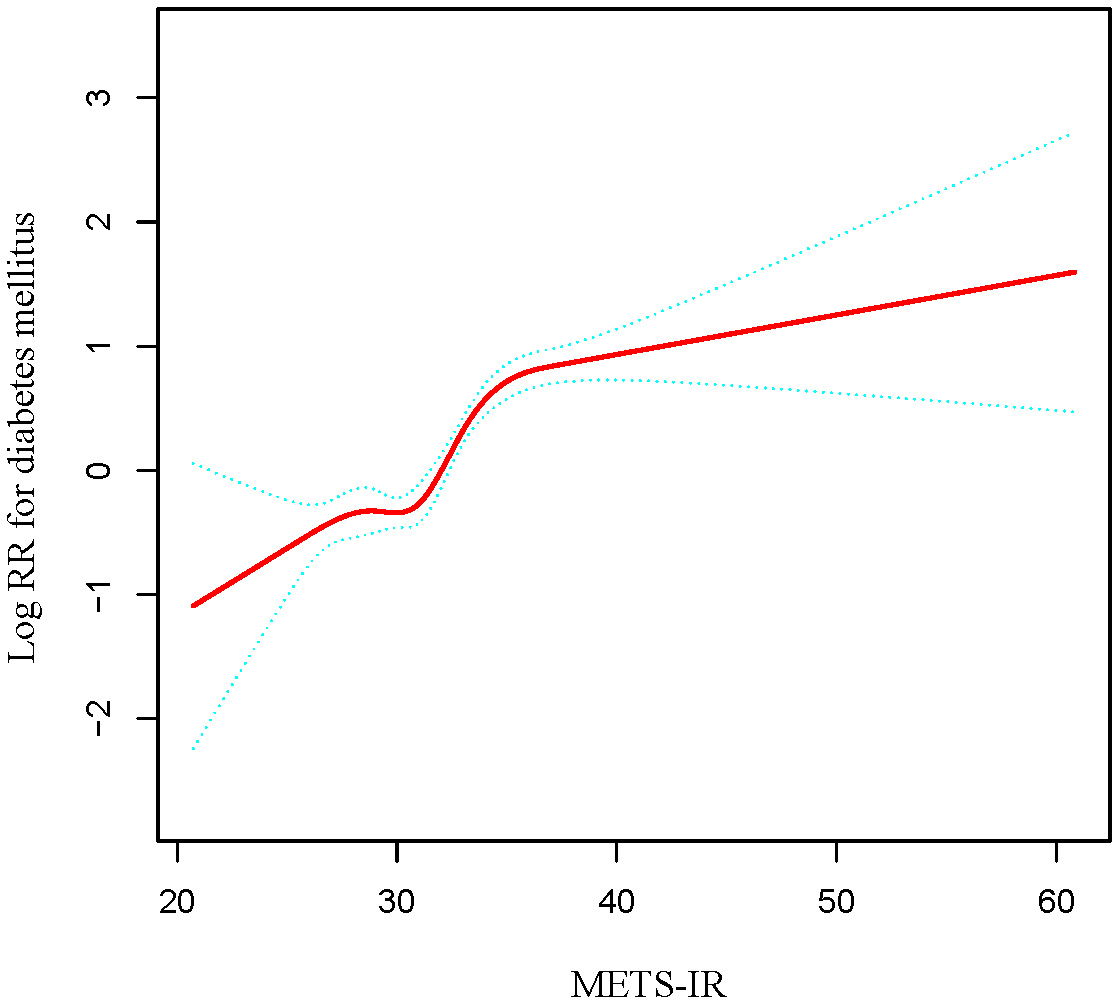
The non-linear relationship between METS-IR and diabetes mellitus in normal-weight middle-aged and older Chinese adults. A non-linear relationship between them was detected after adjusting for gender, age, SBP, DBP, family history of diabetes, TC, LDL-C, Scr, BUN, and ALT. The red curve on the graph represents the smoothed fit between the variables, while the blue lines indicate the 95% confidence intervals surrounding the fit.

The dose-response relationship between METS-IR and diabetes risk was significantly non-linear (*P* < 0.001), characterized by a steep increase in risk below the inflection point of 37.24 (HR 1.18 per unit) that plateaued to non-significance above this threshold (Table 3 and Figure 3).

**Table 3:** Threshold effect analysis between METS-IR and diabetes mellitus in normal-weight middle-aged and older Chinese adults.

**Threshold effect analysis**	**Outcomes**
	**HR (95%CI)** ***p*****-value**
Inflection point of METS-IR (K)	37.24
< K slope	1.18 (1.14, 1.21) < 0.0001
>K slope	0.96 (0.89, 1.04) 0.3494
Log-likelihood ratio test	< 0.001

The above model was adjusted for gender, age, SBP, DBP, family history of diabetes, TC, LDL-C, Scr, BUN, and ALT. Missing data were handled using complete-case analysis. Sample size (*n* = 22,921).

To assess the robustness of the results, sensitivity analyses were performed to address covariates with missing proportions exceeding 5%. First, analyses were conducted by including variables with high rates of missing data (smoking and drinking status) without imputation. Second, all covariates with more than 5% missing values (including smoking and drinking status) were assigned dummy variables, and the models were re-fit accordingly. The non-linear association between METS-IR and DM in normal-weight, middle-aged and older Chinese adults remained evident, confirming the stability of our primary results (Supplementary Tables S1, S2).

### Subgroup analyses

Subgroup analyses explored potential effect modification by age, family history of diabetes, and gender (Table 4). The positive association was consistently observed in all predefined subgroups. The strength of this association appeared to be modified by age (*P* for interaction = 0.0119), with a slightly higher hazard ratio observed in the middle-aged group (45 to < 60 years; HR 1.14, 95% CI 1.11–1.17) compared to the older group (≥60 years; HR 1.09, 95% CI 1.06–1.12). In contrast, no significant effect modification was detected for sex (*P* for interaction = 0.8618) or family history of diabetes (*P* for interaction = 0.5050).

**Table 4:** Subgroup analysis.

**Characteristic**	***N***	**HR (95%CI)**	***P*-value**	***P*-interaction**
Age				0.0119
45≥, < 60	15,652	1.14 (1.11, 1.17)	< 0.0001	
≥60	7,269	1.09 (1.06, 1.12)	< 0.0001	
Gender				0.8618
Male	10,198	1.12 (1.09, 1.14)	< 0.0001	
Female	12,723	1.12 (1.09, 1.15)	< 0.0001	
Family history of diabetes				0.5050
No	22,412	1.12 (1.10, 1.14)	< 0.0001	
Yes	509	1.08 (0.97, 1.20)	0.1668	

The above model was adjusted for gender, age, SBP, DBP, family history of diabetes, TC, LDL-C, Scr, BUN, and ALT; and the model was not adjusted for the stratification variable in each case. Subgroup analyses by smoking and drinking status were not performed due to insufficient data resulting from a high degree of missing values.

## Discussion

This multicenter, retrospective cohort study demonstrated a robust, non-linear, and positive association between METS-IR and incident diabetes in normal-weight middle-aged and older Chinese adults. Following comprehensive adjustment for demographic, clinical, and biochemical confounders, each 1-unit rise in METS-IR corresponded to a 12% increase in diabetes risk. A clear inflection point was observed at a METS-IR of 37.24, below which the risk increased more sharply (HR: 1.18 per unit), while above this point, the association was attenuated and lost statistical significance.

METS-IR serves as a promising non-insulin-based indicator by incorporating critical components such as FPG, BMI, HDL-C, and TG (12). This integrative approach enables a simple and cost-effective assessment of IR, which is particularly useful in large epidemiological studies (27, 28). Our findings in a normal-weight population align with prior studies in mixed-weight and general cohorts, confirming a positive link between METS-IR and diabetes risk (29, 30). Notably, Chen et al. identified a non-linear association between METS-IR and diabetes risk, similar to our findings; however, compared with our METS-IR inflection point of 37.24, theirs occurred at a higher METS-IR level (30). This difference may reflect varying metabolic risk patterns across study cohorts. Our research specifically evaluated normal-weight adults aged ≥45 years (BMI 18.5–23.9 kg/m^2^), a demographic that may exhibit a heightened susceptibility to the effects of IR, even at comparatively lower METS-IR values. Research has indicated that metabolic risk factors exert different influences depending on an individual's age and body composition (24, 31). Moreover, it is plausible that this subgroup presents heightened metabolic vulnerability; factors such as progressive β-cell dysfunction and adverse body composition, despite a normal BMI (including possible sarcopenia or greater visceral adiposity), could exacerbate diabetes risk and allow earlier detection of an inflection point (32, 33). Furthermore, the saturation effect observed beyond the inflection point in our cohort suggests that at higher METS-IR levels, additional pathophysiological processes likely increase diabetes risk. The saturation effect noted beyond this inflection point—where the association with diabetes risk significantly attenuates—emphasizes the need for a nuanced understanding in specific populations. Thus, recognizing these distinctions underscores the importance of population- tailored risk stratification and reinforces the argument that universal cut-off values for metabolic indices, such as METS-IR may not suit all demographic groups (34, 35).

The underlying mechanisms linking METS-IR to the risk of DM, particularly in normal-weight middle-aged and older Chinese adults, may involve several interconnected pathways. Even within the normal BMI range, elevated METS-IR levels may signal ectopic fat accumulation, especially in the liver and muscles, thus thereby impairing insulin signaling and adversely affecting glucose metabolism. This suggests an early onset of diabetes risk, even in ostensibly healthy individuals (30, 34). Dyslipidemia manifests as high TG and low HDL-C levels, hallmark features of IR. These lipid abnormalities indicate a predisposition to metabolic dysfunction regardless of body weight (36, 37). Furthermore, elevated FPG reflects declining glucose regulation, signaling a chronic state of reduced insulin sensitivity. These observations highlight the inadequacy of BMI as a sole health metric. Its inability to capture harmful body composition changes, particularly visceral adiposity and age-related sarcopenia, renders it a misleading indicator, in older adults (38, 39). The relationship between METS-IR and diabetes is not strictly linear; evidence suggests that a metabolic threshold exists below which, incremental increases in METS-IR correspond to a significant rise in diabetes risk, reflecting a progressive deterioration in IR and metabolic stability (40). Once this threshold is surpassed, impaired beta-cell secretion may become the primary limiting factor in diabetes progression, emphasizing the value of early intervention before clinical symptoms appear. These findings underscore the limitations of relying solely on traditional metrics, such as BMI, and instead support broader evaluations of metabolic health, as increased METS-IR levels can precede diabetes diagnosis (41).

METS-IR, as an integrated measure of glucose, lipid, and adiposity parameters, may also be closely related to liver health and vascular structural changes (42). For instance, elevated ALT levels often indicate NAFLD, which is not uncommon in normal-weight individuals—a condition termed lean NAFLD (11, 43). Moreover, an increase in CIMT can also occur in normal-weight middle-aged and elderly individuals who exhibit cardiometabolic risk factors, including central obesity, hypertension, or elevated blood glucose (20). While our study lacked data on NAFLD or subclinical atherosclerosis, the METS-IR components are known correlates of these conditions. Future studies integrating imaging or biomarker data could explore whether the METS-IR-DM relationship is mediated through hepatic or vascular pathways.

Our study has several notable strengths. First, the findings are supported by data from a sizable multicenter cohort in China, ensuring robust and reliable evidence. Second, our investigation focuses on middle-aged and older individuals with normal body weight—a demographic often considered “low-risk” and overlooked in routine metabolic screening. Moreover, our analysis reveals a non-linear positive association between METS-IR and DM within this population, thereby contributing a novel perspective to the existing literature.

Nevertheless, certain limitations should be acknowledged. First, the observational cohort design precludes definitive causal inference between METS-IR and DM. Second, because this study involves a secondary analysis of a publicly available dataset, data on NAFLD and CIMT were unavailable, restricting comprehensive evaluation. Third, as the evidence stems from health examination data of a Chinese cohort, caution is warranted when extrapolating these findings to other populations.

Finally, despite adjustments for known confounders, this observational study cannot eliminate potential residual bias from unmeasured factors, such as diet and physical activity. Most critically, A major limitation is the very high rate of missing data for key lifestyle confounders (smoking, drinking). This prevents adequate control for these factors and limits the interpretability of models that include them.

Based on the novel demonstration of a non-linear relationship between METS-IR and diabetes risk, with a distinct threshold at METS-IR = 37.24 in normal-weight middle-aged and older Chinese adults, we recommend that researchers: (i) initiate prospective studies to establish causality between METS-IR and diabetes incidence, (ii) clarify dynamic changes in insulin sensitivity and β-cell compensation underlying the observed METS-IR threshold, and (iii) enhance integration of this metric into precision–prevention frameworks to address gaps in current metabolic risk assessment for normal-weight individuals, (iv) validate the identified METS-IR threshold (37.24) in independent, ethnically diverse cohorts of normal-weight individuals.

## Conclusions

This large cohort study identified a significant non-linear relationship between METS-IR and DM risk among normal-weight middle-aged and older Chinese adults, with a threshold effect around 37.24 below which the risk increased markedly. These findings highlight the potential of METS-IR to improve early risk stratification and inform prevention strategies in this population.

## Data Availability

The datasets presented in this study can be found in online repositories. The names of the repository/repositories and accession number(s) can be found below: https://datadryad.org/stash/dataset/doi:10.5061%2Fdryad.ft8750v.
