# Supplementary material for: Non-linear association between metabolic score for insulin resistance and diabetes mellitus in normal-weight middle-aged and older Chinese adults: a multicenter retrospective cohort study
Source: Front Nutr. 2026 Jan 20;12:1717792. doi: 10.3389/fnut.2025.1717792 (PMC12864058; doi:10.3389/fnut.2025.1717792)
Supplement: Supplementary file 1 [file Table_1.docx]

Supplementary Material

# Table S1 Threshold effect analysis between METS-IR and diabetes mellitus in normal-weight middle-aged and older Chinese adults

| Threshold effect analysis | Outcome |
| --- | --- |
|  | HR (95%CI)*p*-value |
| Inflection point of METS-IR (K) | 37.75 |
| <K slope | 1.18 (1.12, 1.25) <0.0001 |
| >K slope | 0.97 (0.84, 1.12) 0.6915 |
| Log-likelihood ratio test | 0.007 |

The above model was adjusted for gender, age, SBP, DBP, family history of diabetes, drinking status, smoking status, TC, LDL-C, Scr, BUN, and ALT. Missing data were handled using complete-case analysis. Sample size (n = 5589).

# Table S2 Threshold effect analysis between METS-IR and diabetes mellitus in normal-weight middle-aged and older Chinese adults

| Threshold effect analysis | Outcome |
| --- | --- |
|  | HR (95%CI)*p*-value |
| Inflection point of METS-IR (K) | 37.24 |
| <K slope | 1.18 (1.14, 1.21) <0.0001 |
| >K slope | 0.96 (0.89, 1.04) 0.3563 |
| Log-likelihood ratio test | <0.001 |

The above model was adjusted for gender, age, SBP, DBP, family history of diabetes, drinking status, smoking status, TC, LDL-C, Scr, BUN, and ALT. Dummy variables were employed to indicate missing covariates values when the proportion of missing values exceeded 5%. Sample size (n = 22921).
